# Guidelines vs mindlines: a qualitative investigation of how clinicians’ beliefs influence the application of rapid molecular diagnostics in intensive care

**DOI:** 10.1128/aac.01156-24

**Published:** 2025-02-05

**Authors:** Sarah-Jane F. Stewart, Alyssa M. Pandolfo, Yogini Jani, Zoe Moon, David Brealey, Virve I. Enne, David M. Livermore, Vanya Gant, Stephen J. Brett, Rob Horne, the INHALE WP4 Study Group, Julie A. Barber

**Affiliations:** 1 School of Pharmacy, University College London 371646 , London, United Kingdom; 2 UCLH-UCL Centre for Medicines Optimisation Research and Education, University College London Hospitals NHS Foundation Trust 8964 , London, United Kingdom; 3 Division of Critical Care, University College London Hospitals NHS Foundation Trust 8964 , London, United Kingdom; 4 Division of Infection and Immunity, University College London Faculty of Medical Sciences 308348 , London, United Kingdom; 5 Norwich Medical School, University of East Anglia 12201 , Norwich, United Kingdom; 6 Department of Medical Microbiology, University College London Hospitals NHS Foundation Trust 8964 , London, United Kingdom; 7 Department of Surgery and Cancer, Imperial College London 170714 , London, United Kingdom; Johns Hopkins University School of Medicine, Baltimore, Maryland, USA

**Keywords:** rapid molecular diagnostics, antibiotic prescribing, intensive care, pneumonia

## Abstract

Rapid molecular diagnostic tests improve antimicrobial stewardship (AMS) by facilitating earlier refinement of antimicrobial therapy. The INHALE trial tested the application of the BioFire FilmArray Pneumonia Panel (Pneumonia Panel) for antibiotic prescribing for hospital-acquired and ventilator-associated pneumonias (HAP/VAP) in UK intensive care units (ICUs). We report a behavioral study embedded within the INHALE trial examining clinicians’ perceptions of using these tests. Semi-structured interviews were conducted with 20 ICU clinicians after using the Pneumonia Panel to manage suspected HAP/VAP. Thematic analysis identified factors reinforcing perceptions of the necessity to modify antibiotic prescribing in accordance with test results and doubts/concerns about doing so. While most acknowledged the importance of AMS, the test’s impact on prescribing decisions was limited. Concerns about potential consequences of undertreatment to the patient and prescriber were often more salient than AMS, sometimes leading to “just-in-case” antibiotic prescriptions. Test results indicating a broad-spectrum antibiotic were unnecessary often failed to influence clinicians to avoid an initial prescription or de-escalate antibiotics early as they considered their use to be necessary to protect the patient and themselves, “erring on the side of caution.” Some clinicians described cases where antibiotics would be prescribed for a sick patient regardless of test results because, in their opinion, it fits with the clinical picture—“treating the patient, not the result.” Our findings illustrate a tension between prescribing guidelines and clinicians’ “mindlines,” characterized by previous experiences. This highlights the need for a “technology plus” approach, recognizing the challenges clinicians face when applying technological solutions to patient care.

**IMPORTANCE:**

Rapid molecular diagnostic tests for pathogens and resistance genes may improve antibiotic-prescribing decisions and stewardship. However, clinicians’ desire to protect their patients with antibiotics often overrides more distal concerns about possible resistance selection, limiting the application of these tests in practice. Findings underscore the challenge of changing prescribing decisions based on technical results or guidelines, highlighting factors such as clinicians’ previous experience and “knowledge in practice” as more proximal drivers of these decisions. Implementation strategies for technological solutions to antimicrobial resistance must be “behaviorally intelligent,” recognizing the challenges facing clinicians when making “life or death” prescribing decisions.

**CLINICAL TRIALS:**

This study is registered with ISRCTN as ISRCTN16483855.

## INTRODUCTION

Antibiotic prescribing is challenging and complex, particularly in intensive care units (ICUs) where diagnostic uncertainty coupled with high-stakes consequences is the norm. Antibiotics can have undesirable effects such as adverse drug reactions and promotion of *Clostridium* (*Clostridioides*) *difficile* infection (1); more generally, the overuse of broad-spectrum antibiotics drives the selection of antimicrobial resistance (AMR) most notably in the patient’s gut flora (2). On the other hand, initial empirical cover may be inadequate for patients infected with unusually drug-resistant bacteria (3).

There is increasing interest in the use of rapid molecular microbiology diagnostic tests. These have the potential to improve antimicrobial stewardship (AMS) by rapidly identifying the type of infecting organism and specific agents to which it is likely to be resistant. In principle, this should enable clinicians to avoid prescribing an unnecessary broad-spectrum antibiotic or stop one early if test results suggest that a narrower-spectrum agent is adequate to combat the particular pathogen(s) found. The FilmArray (4) and Unyvero (5) tests can detect multiple respiratory pathogens and antimicrobial resistance genes directly from respiratory secretions, with results in 1–6 hours compared with current, culture-based, turnarounds of 48–72 hours (6). Moreover, pathogens are found in a greater proportion of samples than by conventional microbiology (7, 8).

One area where rapid molecular microbiology diagnostic tests are being evaluated is in the treatment of patients with suspected hospital-acquired and ventilator-associated pneumonias (HAP/VAPs) in ICUs. HAP/VAPs are common in these units, necessitate urgent antimicrobial therapy (9), and have substantial mortality (10, 11). Current best practice for suspected HAP/VAP patients is the initial prescribing of empiric broad-spectrum antibiotics, covering all likely pathogens, with later refinement once laboratory culture results become available, typically in 48–72 hours (9). Although this approach is well-established, it has considerable limitations. First, HAP/VAPs can be challenging to diagnose without laboratory culture because ICU patients can exhibit signs suggesting bacterial pneumonia even in its absence (12, 13). Furthermore, as many as 70% of patients with clinically diagnosed pneumonia have no pathogen grown in laboratory cultures (5). Because their pathogen(s) remain unspecified, such patients cannot have their treatment refined and often remain on broad-spectrum agents for prolonged periods. Combined, these factors may result in a greater use of broad-spectrum antibiotics than necessary (2). The application of molecular diagnostics in the treatment of HAP/VAP in ICU settings is currently being investigated through randomized controlled trials (RCTs). These trials are investigating the utility of multiplex PCR tests such as the BioFire FilmArray Pneumonia Panel (bioMérieux) (the “Pneumonia Panel” test) (4) and Curetis Unyvero Hospitalized Pneumonia cartridge (7, 14). One example in the United Kingdom is INHALE (15), which is examining the accuracy of these tests and their influence on AMS and clinical outcomes.

The future implementation and adoption of these tests are likely to be substantially driven by clinicians’ perceptions (2, 16, 17), but there are limited data available on how these technologies may influence future prescribing behavior. For this reason, a series of behavioral studies were embedded within INHALE to explore clinicians’ perspectives on antibiotic prescribing for HAP/VAP and their perceptions of the role and potential of molecular diagnostics. The first study was initiated before the trial and examined clinicians’ attitudes to prescribing antibiotics for HAP/VAP, how they judged the necessity for broad-spectrum antibiotics for individual patients, and how they balanced these necessities against concerns about AMS (2). A further pre-trial study explored clinicians’ attitudes and perceptions of applying rapid molecular microbiology tests for HAP/VAP (16). Although clinicians were concerned about AMR and perceived these tests to be of potential value in supporting antimicrobial prescribing and stewardship, they had concerns about their application in clinical practice, particularly regarding unfamiliarity with the tests’ capabilities and a lack of confidence in “negative” results. These studies showed that the Necessity Concerns Framework (NCF) (18) could be applied to understand clinicians’ perspectives on antibiotic prescribing. They also identified potential barriers to the implementation of molecular diagnostics in practice. Furthermore, they informed the design of the present study, which aimed to explore clinicians’ perspectives and decision-making when using Pneumonia Panel tests as a prescribing decision aid for intervention-arm HAP/VAP patients participating in the INHALE RCT (19).

## MATERIALS AND METHODS

This research is part of the INHALE research program (ISRCTN16483855) (20), funded by the National Institute for Health Research, investigating the utility of molecular diagnostics to guide antimicrobial prescribing for ICU patients with suspected HAP/VAPs. INHALE includes a RCT whereby HAP/VAP patients at 14 ICUs were randomized to (i) standard empirical antibiotics, adapted once routine microbiology results become available, or (ii) initial antibiotic therapy guided by a point-of-care (POC) rapid molecular diagnostic (the FilmArray Pneumonia Plus Panel–the Pneumonia Panel test) (4), with this treatment adapted once routine microbiology results become available (19). Clinicians treating intervention-arm patients could use a locally approved prescribing algorithm that recommended, but did not mandate, possible antibiotics appropriate to particular molecular diagnostic results. The Pneumonia Panel uses multiplex polymerase-chain reactions (PCR) to seek pathogens and their resistance genes (Table S1). It was chosen for the RCT following head-to-head evaluation with the Curetis Unyvero Hospitalized Pneumonia Cartridge; this evaluation considered pathogen detection accuracy, speed, ease of use, and reliability (7).

Research Ethics Committee approval was obtained from the London-Brighton & Sussex Research Ethics Committee (19/LO/0400) before data collection, and this article was written following Standards for Reporting Qualitative Research guidelines (File S2) (21).

### Participants

To be eligible for interview, clinicians had to be practicing in one of the 14 UK ICUs participating in the INHALE RCT (Table 1). Furthermore, participants needed to have experience using Pneumonia Panel results to guide an antibiotic decision for at least one INHALE intervention-arm patient. Participants were identified and recruited by A.M.P., V.I.E., D.B., V.G., and the sites’ research nurses. Research nurses had a log of all clinicians who met the above eligibility criteria, all of whom were then invited to participate via email. Interviews were conducted when clinicians were not working.

**TABLE 1:** Hospital and participant characteristics

Hospital no.	Location in the United Kingdom	Hospital type	Clinician role
1^*a*^	London	Teaching hospital	Four ICU consultants One consultant clinical microbiologist
2	London	Teaching hospital	One ICU consultant
3	Liverpool	Teaching hospital	Two ICU consultants
4	Hertfordshire	District general hospital	Two ICU consultants
5^*b*^	Birmingham	Specialist pediatric hospital	Two ICU consultants
6	London	Teaching hospital	One ICU consultant Two consultant clinical microbiologists
7	Liverpool	Teaching hospital	One ICU consultant
8	Stoke-on-Trent	Teaching hospital	One ICU consultant
9	London	Private hospital	One ICU consultant
10	London	Specialist pediatric hospital	One ICU consultant^*c*^ One consultant clinical microbiologist

^*a*^ Patients from Hospital 1 comprised approximately a quarter of patients participating in the INHALE randomized controlled trial; we therefore purposively over-sampled clinicians from this hospital to interview a similar proportion of clinicians.

^*b*^ All clinicians from Hospitals 5 and 10 treat pediatric patients; the remainder treat adults.

^*c*^ During the COVID-19 pandemic, this consultant treated adult patients at Hospital 1.

All participants provided written informed consent and were included in the presented analysis.

### Data collection

Interviews were conducted by A.M.P. between August 2020 and May 2021 via Microsoft Teams. Interview durations ranged from 11 to 46 minutes. Semi-structured interviews were conducted with clinicians to explore their perceptions of using the Pneumonia Panel test as a prescribing decision aid for INHALE intervention-arm HAP/VAP patients. Clinicians were asked about a time when they had used Pneumonia Panel results to guide an antibiotic decision and were asked about barriers and facilitators to incorporating test results into their prescribing decision-making. They were also asked about their experiences of using, and perceptions about, the INHALE trial prescribing algorithm; however, those data are outside the scope of the current research question and hence are not reported here (see File S3 for interview guide).

Interviews were conducted and analyzed concurrently to determine data saturation, which we defined as three interviews eliciting no novel findings (22). It should be noted that the study period included the winter 2020/2021 wave of the COVID-19 pandemic, largely driven by the alpha variant.

### Data analysis

Interviews were recorded, transcribed verbatim, and anonymized by A.M.P. and Y.J. (consultant pharmacist). For reflexivity (23), our team has previously conducted qualitative and quantitative research on ICU clinician antibiotic decision-making and attitudes toward rapid diagnostics; however, we strove to remain neutral and data-driven during analyses (2, 16).

Braun and Clarke’s (24,24) recommendations for deductive thematic analysis were followed, applying the NCF (18). Our previous published work (2, 16) outlines how the NCF can be applied to clinicians’ perspectives surrounding their antibiotic-prescribing decision-making, highlighting that when making decisions, clinicians weigh up their perceptions of the necessity for antibiotics/rapid diagnostic test against their concerns. This approach was carried forward into the present analysis when applying the NCF to the interview transcripts.

An interpretivist approach was applied to understand clinicians’ beliefs about using the Pneumonia Panel as a prescribing decision aid (25). A.M.P. first coded the transcripts in NVivo (version 12) at the semantic level, summarizing content explicitly discussed by multiple participants reflecting clinicians’ beliefs about using the Pneumonia Panel test and other contextual factors perceived to influence their use of the test (26). When grouping codes, a deductive approach was used, applying the NCF to construct two pre-conceived themes reflecting beliefs about the importance (necessity) of, and doubts/concerns about, applying the test: (i) “Factors reinforcing the necessity to modify antibiotic prescribing in accordance with rapid molecular test results” (i.e., ICU clinicians’ perceptions of the importance of the molecular microbiology results in practice) and (ii) “Doubts about the necessity to modify antibiotic prescribing in accordance with rapid molecular test results” (i.e., ICU clinicians’ concerns about the challenges associated with applying the test in clinical practice) (27). Similar codes within each of the two themes were then grouped together to form subthemes (e.g., a pattern of specific concerns about applying the Pneumonia Panel). Following Braun and Clarke’s recommendations, thematic maps were created to organize, develop, and visualize the analysis, which evolved iteratively until a final thematic map was created. Only data relevant to the clinicians’ beliefs about the molecular diagnostic tests are represented in the present analysis.

Y.J. provided support to A.M.P. throughout the analytic process by listening to interview recordings and reading transcripts to discern unclear communication. To ensure analytic quality, the analysis was sense-checked at multiple stages with Y.J., R.H., S.B., D.B., and other INHALE collaborators. Interviews and data analysis were conducted concurrently to determine data saturation, when no new themes or subthemes were created from additional interviews.

## RESULTS

Participants comprised 20 clinicians working in 10 of the 14 English ICUs participating in INHALE. Sixteen were consultants in intensive care medicine and four were consultant clinical microbiologists (Table 1).

“Factors reinforcing the necessity to modify antibiotic prescribing in accordance with test results” (four sub-themes) are described first, followed by “Doubts about the necessity to modify antibiotic prescribing in accordance with rapid molecular test results” (nine sub-themes). Sub-themes and supporting quotations for “Factors reinforcing the necessity to modify antibiotic prescribing in accordance with rapid molecular diagnostic test results” and “Doubts about the necessity to modify antibiotic prescribing in accordance with rapid molecular diagnostic test results” themes are presented in Tables 2 and 3, respectively.

**TABLE 2:** Factors reinforcing the necessity to modify antibiotic prescribing in accordance with rapid molecular test results^*a*^

Sub-theme	Quote number	Supporting quotations
Rapidity of results enabled earlier refinement of antimicrobial therapy	1	“One of the frustrations I have as an intensive care consultant is the turnaround times for most microbiological tests, which frequently lags behind my decision-making. […] you've done what you would normally do for the first day or two and you see the patient not getting any better. And then you’re thinking maybe there’s something I'm missing. Maybe… And to send off another barrage of tests will take another three to four days to come back. You want a rapid [molecular diagnostic] result; you want that patient to get better quickly.” -P14, ICU consultant, Hospital 4
	2	“[Culture result] takes 48 hours, it’s very easy to be lazy and just keep the antibiotics going for longer. Whereas this [molecular test], because it’s immediately available, actually makes you think critically about your clinical decision-making just as the patient’s come in.” -P11, ICU consultant, Hospital 5
Results increase prescribing confidence under clinical uncertainty	3	“You do a [molecular diagnostic] test when you’re worried about something [i.e., infection]. And obviously [if] further tests show something that ties in with your clinical gestalt, as it were. You then can treat, and if it reassures, you know, it can be a rule in or rule out. And you know, and it might be a comfort blanket, you know. I don’t want to treat, and the test shows me there’s nothing to treat so therefore it reinforces my confidence level. […] a huge number of people are treated inappropriately [with antibiotics]. But the problem is they’re doing it just in case rather than, you know, having sound microbiological proof.” -P15, ICU consultant, Hospital 1
	4	“[Antibiotic decisions] are life or death decisions. And these decisions are probability decisions. And definitely you need to cover every single possibility because sometimes you might be wrong. And the margin for error in a patient who is on an acute critical illness, multi-organ failure is minimal. […] [molecular diagnostics help] make a better decision, or better in terms of probability. Because anyway, a decision can be wrong even having all the probabilities because you will need to choose. And what may be chosen [may] not necessarily [be] the right decision sometimes. But at least you might be in a position to argue that your prescription or your behavior in the way you prescribe it was based on signs and not just pure speculation or pure gut feeling.” -P6, ICU consultant, Hospital 9
	5	“[Molecular test] gives an extra piece of information in that puzzle, as it were, to help you decide should I, shouldn’t I treat […] It should not be used in isolation.” -P15, ICU consultant, Hospital 1
Positive results were valuable in supporting antibiotic choice and stewardship	6	“[Molecular diagnostic results are] useful to confirm infection, provide some guidance about antibiotics […] [It detected] a Gram-negative *Enterobacteriaceae*, which may have had a CTX-M gene indicating it was an ESBL producer. And that would guide us toward an antibiotic active against that. So temocillin or ertapenem, rather than Tazocin or Augmentin. […] Another example, I think, would be when there was a *Pseudomonas* detected. Where we haven't seen a *Pseudomonas* in that patient before, and so that would guide us toward including [an] antipseudomonal antibiotic in the treatment.” -P9, consultant microbiologist, Hospital 6
	7	“we would score someone for a VAP. Based on their chest X-ray changes, do they have increased amount of sputum, is their white cell count up, do they have a temperature? And do they then grow any organisms? So, if you put it into a point-scoring system and then if you know they’re growing organisms from the BioFire, then you would treat [with antibiotics].” -P2, ICU consultant, Hospital 3
	8	“the [molecular test] result came back. It was getting *Proteus* in the… in the tracheal aspirate sample […] [without molecular diagnostics] probably wouldn’t have used ceftriaxone. I doubt we would have used… It may have been Tazocin but like some of my colleagues love Tazocin. But it would… probably it so… it might, but it may even have been meropenem based on the [microbiology] recommendation. So, the BioFire enabled a narrower spectrum antibiotic.” -P1, ICU consultant, Hospital 3
Results aid differential diagnosis for patients with COVID-19	9	“what was, you know, COVID-driven inflammatory blood resistant [white] count and what we could see whether there was an indication that this is a bacterial pneumonia. Because the chest X-rays were equally awful in patients who had bacterial pneumonia and who didn’t. And in addition, it was complicated by the fact that patients then in the middle of the first wave and then in the second wave were given drugs that would affect inflammatory markers. […] if the BioFire was negative, yes there might be a moderation of his… anti-infectives after 24/48 hours, but actually they [clinicians] were using it as an insight whether they would give this patient high dose steroids.” -P19, consultant microbiologist, Hospital 6
	10	“[a] COVID patient who deteriorates, we’ve probably got to make a decision within 24 or 48 hours. Is this rip roaring [i.e., serious] infection that needs to be treated and therefore don’t suppress their immune system anymore? Or, on the other hand, is this immune system gone mad because of the COVID? In which case we suppress the immune system, which would be entirely the wrong thing to do if they’ve got [an] infection.” -P7, ICU consultant, Hospital 7
	11	“Patients [with COVID-19] would get a lot of empirical antibiotics. So that’s probably a circumstance where having a negative BioFire might just provide more evidence that really there was no ongoing bacterial infection. And no benefit from the empirical antibiotics, so [it would] help with stopping and antibiotic stewardship there. Because most of the patients with COVID didn’t have a bacterial infection. Certainly initially. And then over time they… they did get bacterial infections over time as they were in the intensive care unit for longer. And then the same, reverse would apply if we started empirical antibiotics and got a positive result on BioFire. That could help tailor antibiotic treatment sooner than the conventional cultures.” -P9, consultant microbiologist, Hospital 6

^*a*^ BAL, bronchoalveolar lavage; BioFire, BioFire FilmArray pneumonia panel; DNA, deoxyribonucleic acid; ESBL, extended spectrum beta-lactamase; ICU, intensive care unit; Tazocin, piperacillin/tazobactam; and VAP, ventilator-associated pneumonia.

**TABLE 3:** Doubts about the necessity to modify antibiotic prescribing in accordance with rapid molecular test results^*a*^

Sub-theme	Quote number	Supporting quotations
Treating the patient not the result	1	“Um I would always treat the patient and not the result. So regardless of what type of sample analysis has been used, I would treat the patient, so if I felt the patient had clinical features of infection, I would treat them for infection unless I felt it was going to be harmful to do so.” *-*P8, ICU consultant, Hospital 7
	2	“If the patient’s super sick, I don’t care what the test says, I’m prescribing antibiotics because, you know, that fits with the clinical picture. If the patient is super well and… and the… and the test result doesn’t corroborate with all the other evidence that I’m triangulating, because no one test is perfect, I’m not going to prescribe antibiotics.” *-*P20, ICU consultant, Hospital 1
	3	“If the organism did not… was not detected, but there was a clinical suspicion for ventilator-associated pneumonia, we would carry on with the antibiotics anyway.” *-*P10, ICU consultant, Hospital 8
	4	“I have to say as a clinician I don’t follow guidelines very well. I tend to go by my gut instinct and by what I see by the patient’s physiology, by the bed space. And frequently, even if the guidelines suggest a different antibiotic, sometimes I change my… my plans. Not on the basis of either the BioFire or… or… It’s all in the whole kind of holistic view about what’s going on.” -P14, ICU Consultant, Hospital 4
Negative results create dilemmas	5	“a negative [molecular diagnostic] test, if it’s well performed, is trying to say to you we cannot identify any bacterial DNA. […] we’ve got no evidence that there is some sort of… one of the common pathogens here, in that [sample]. And so that’s sort of saying to you: Look, you’ve got little evidence to support active infection.” -P4, ICU consultant, Hospital 1
	6	“[I’ve] chosen to stop them [antibiotics] as a result of the negative BioFire result. So, in a sense, saving two or three days or potentially more of an antibiotic. We do… and I've just seen that as an example in my own mind of good practice, you know, good antibiotic stewardship.” -P3, ICU consultant, Hospital 2
	7	“We refocused the antibiotics on sepsis rather than chest sepsis. So, the antibiotics were not stopped, but the BioFire… the results of the BioFire were negative...” -P11, ICU consultant, Hospital 5
Initial skepticism and unfamiliarity	8	“So that even if it’s an antibiotic we’re unfamiliar [with], we don’t routinely use like ceftriaxone for pneumonia, we only tend to use it for things like CNS infection […] 'Cause usually it’s unfamiliarity. It’s the situation where [changes voice] “We don’t normally do this, so I don’t want to do it”, which is how a quite a few of my colleagues still practice. […] I think initially there’s a degree of skepticism because again, the department, well most departments I suspect is slightly split between people who are interested in new things and people [who] are not really that bothered by new things. And I think it was a little bit split.” *-*P1, ICU consultant, Hospital 3
	9	“Most intensive care doctors come with a healthy streak of skepticism about a new machine. Is it really going to add something that’s going to change practice?” -P16, ICU consultant, Hospital 10
	10	“I haven’t used it [molecular diagnostics] enough […] I really would need more involvement with it.” *-*P17, ICU consultant, Hospital 1
	11	It’s a matter of exposure. So if you have the machine and you use the machine, finally you are used to make decisions with that information. If that machine is available, but it’s not integrated in their routine because you have few patients [that] makes you less confident of using information from the machine. I think it’s a matter of exposure, is… is… is not a… the machine are such that is triggering your decision or your confidence with devices. If something is integrated that it’s part of a pathway and you’ve got enough volume of patients to… to be exposed to… to that pathway decision, definitely you will have an opportunity to be more confident with the machine. So I think it’s not the machine as such. It’s how much this machine is using the context of making decisions.” *-*P6, ICU consultant, Hospital 9
Variable knowledge of the tests’ inherent limitations	12	“[Molecular diagnostic tests] can’t distinguish between live or dead bacteria, but well, that’s not a concern. That’s like a feature of understanding how the tools you have available to help you, work. And like there isn’t a perfect tool. So, it’s just a piece of knowledge that yeah, you need to have while you’re doing these things.” -P4, ICU consultant, Hospital 1
	13	“I can’t remember if *Stenotrophomonas* was on there [panel]. I think it… maybe it wasn’t, I don’t know. And we’ve had *Elizabethkingia* bacteria.” -P12, ICU consultant, Hospital 5
	14	“[Patients] can’t really be on antibiotics, you wouldn’t use it [molecular diagnostic test] then.” -P17, ICU consultant, Hospital 1
Respiratory sample unavailability and of uncertain quality	15	“the COVID patients, they were just dry [as] a bone. You could never get specimen once they’d been there [ICU] for 3–4 days. […] You can’t do the test if you haven’t got sputum. So. And these patients are on a lot of oxygen, so you’re not inclined to do bronchoalveolar lavages on them either.” -P13, ICU consultant, Hospital 4
	16	“[In the COVID-19 surges] the ICU staff may not have been familiar with procedures in an ICU in general, let alone what a BioFire is. Particular locations meant that it was more difficult for the research nurses to have time to go and consent a patient and also pick up a sample in a, sort of, in a timely fashion […] there was competing workload from other trials that were running, on the research nurses. So, when you combine all of those three, a quite common event would be that we would identify somebody on the ward round who would meet the criteria to be recruited, but they weren’t. So, they would have a sample sent for MC&S [microbiology culture & sensitivity], but they didn’t get a sample taken for BioFire.” -P19, consultant microbiologist, Hospital 6
	17	“I don‘t know how good our quality control was for sampling. […] nurse or research nurse or physio, whoever is collecting samples, [do] they apply anything like the same kind of quality control to ‘That’s proper sample, and that isn’t’?” -P16, ICU consultant, Hospital 10
	18	“[The molecular diagnostic test is] on a desktop in intensive care or something like that. Where they’re less used to handling sensitive PCR machines. And has the potential to be contaminated by bugs and flora.” -P18, consultant microbiologist, Hospital 10
	19	“deep [BAL-like] sampling is a better… in a sense is closer to the ‘truth’, if you like, in inverted commas, about pneumonia. As opposed to proximal [sputum-like] sampling. And so of course, a lot of these patients had proximal sampling. And so, were we actually just dealing with colonization?” -P3, ICU consultant, Hospital 2
	20	“it’s easy to mess up a BAL so the test comes back negative.” -P20, ICU consultant, Hospital 1
False-positive results encouraging antibiotic overtreatment	21	“the temptation for the [ICU] clinician is to try and treat all of those organisms [detected by molecular diagnostics]. Which often mean[s] meropenem […] [intensivists] will be less critical than I am of the results, or if they see a result they will say: ‘Right, what do we give to treat it?’ They won‘t think: ‘Do we need to treat it?’” -P5, consultant microbiologist, Hospital 1
	22	“I need a quantitative assay as opposed to [a] qualitative assay. So, I'm happy to say that well that’s *Klebsiella* in sputum. Fine. But is that *Klebsiella,* is it significant? And that level of significance is what I need.” -P14, ICU consultant, Hospital 4
	23	“where I struggle a bit is to understand what the quantitative piece [of molecular diagnostic results] means.” -P4, ICU consultant, Hospital 1
False-negative results leading to antibiotic undertreatment	24	“if the BioFire is negative and you are still having [a] small possibility that the patient is having [an] infection. Very small possibility, but you might start treatment with antibiotics while you do other things that might not be related with the sepsis. Uhm, you might see that response over the next 24 hours, 48 hours […] [if] the patient dies or have [*sic*] any complication related with an infection and you did not cover that because you restrained yourself, rightly or wrongly, at that particular time. You might see the situation as a potential litigation problem.” -P6, ICU consultant, Hospital 9
	25	“a false negative may give you the confidence to stop therapy when actually they’re [patient] still unwell.” -P18, consultant microbiologist, Hospital 10
	26	“I don't mind false positives 'cause I'll just treat for a while. Um, that’s not… not such a negative, but the false negative would be the thing I don't want to miss.” -P12, ICU consultant, Hospital 5
	27	“there have been a few situations where we’ve not believed a negative result. […] And we've repeated it [molecular test]. And done it with deep [BAL-like] samples and there’s been a… just because of the clinical situation. And we've re-calibrated the machine.” -P3, ICU consultant, Hospital 2
Concerns about how results influence existing AMS structures and communications	28	“if you just use it [molecular diagnostics] on everybody without making a decision beforehand of ‘Do I think they have an infection or not’, you're probably going to end up with a lot of people [getting antibiotics].” -P1, ICU consultant, Hospital 3
	29	“there’s a limit on the number of the test you can run concurrently. I think that that limits… you know, it’s not for all comers [into ICU] as it were. And I think if people start abusing it then you're gonna have patients that you need the results [for] and you’re not gonna get [them].” -P13, ICU consultant, Hospital 4
	30	“[Molecular diagnostic] results come out at 8:00 o’clock [at] night. I don't know why that is particularly, but that’s quite common. […] Sometimes I'm notified and don’t see it till the following morning. So, in the interim, you’ll tend to find they [patients] get put on whatever they [intensivists] think is going to cover it [detected organism].” -P5, consultant microbiologist, Hospital 1
	31	“I wrote on the drug chart the result of the BioFire. So, right next to where the antibiotics are with the box on the day after to say ‘Let’s review this’. So, I was giving a plan and clearly labeling it, but that doesn't mean that it got through to the microbiologists […] if you could find a way to get that result onto our in-house system and flagged to the microbiologist paired up with the BAL sample, then I think that would be really useful.” -P12, ICU consultant, Hospital 5
	32	“one thing that we could have done would have been a way to, you know, scan or image the result and incorporate it into our clinical notes so that it would be apparent to other colleagues why a patient was de-escalated from a carbapenem to temocillin a week ago.” -P19, consultant microbiologist, Hospital 6
Uncertainty about the evidence base for molecular diagnostic test results’ clinical usage	33	“[Molecular diagnostic tests] gotta really show an impact for it to be worth the hassle and the maintenance and the cost and the variability […] It’s gotta be clearly better for it to be adopted.” -P16, ICU consultant, Hospital 10
	34	“[Recruiting in COVID has] been good in that many of my colleagues because we were using off… just using it [molecular diagnostics] routinely, liked it, and gained confidence in it.” -P1, ICU consultant, Hospital 3
	35	“So the BioFire was negative for any of the common organisms. I guess the thing that influenced me is that I didn‘t stop the antibiotic at that time. I decided to continue them over the first 24 hours. For the reasons I’ve already sort of talked about that I haven't built the confidence in the test yet and I haven’t seen the sort of large validated study yet.”*-*P12, ICU consultant, Hospital 5
	36	“Most clinicians would want to know how accurate is that [molecular diagnostic test] and is it inferior or non-inferior? And we would pour over the evidence for that in some detail.” -P7, ICU consultant, Hospital 7

^*a*^ BAL, bronchoalveolar lavage; BioFire, BioFire FilmArray pneumonia panel; ICU, intensive care unit; and PCR, polymerase chain reaction.

### Factors reinforcing the necessity to modify antibiotic prescribing in accordance with rapid molecular test results

#### Rapidity of results enabled earlier refinement of antimicrobial therapy

Many clinicians described the standard care for a patient with suspected HAP/VAP to be the “initial prescribing of broad-spectrum antibiotics, then refining therapy after circa 48–72 hours, once laboratory culture results were received.” The delayed availability of culture results was described as problematic, and Pneumonia Panel test results were perceived to enable pathogen-based antibiotic decisions to be made earlier (i.e., after a few hours compared to days) (Table 2, Quote 1). Participants often described how Pneumonia Panel results were used in combination with other available evidence (e.g., inflammatory markers in blood tests) to make an earlier, better-informed prescribing decision (Table 2, Quote 2).

#### Results increase prescribing confidence under clinical uncertainty

Many reported that antibiotic decision-making was most challenging under conditions of clinical uncertainty—where confidence in a microbiological diagnosis was low (Table 2, Quote 3). In uncertainty, clinicians were concerned about the possible consequences of antibiotic undertreatment for the patient (e.g., an increased risk of mortality) and clinician (e.g., distress and regret at losing the patient, and risk of litigation). Clinicians acknowledged that broad-spectrum antibiotics were often prescribed and continued as a protective measure “*just-in-case”* of infection requiring an antibiotic (Table 2, Quotes 3–5).

In some cases, Pneumonia Panel results increased clinicians’ confidence in the prescription, particularly when these results corroborated the patient’s clinical picture and other test results. One clinician likened having Pneumonia Panel results to a “*comfort blanket”* (Table 2, Quote 3). Some clinicians valued the Pneumonia Panel results as providing assurance for their empirical prescribing, which otherwise relied on what was acknowledged to be “*pure speculation”* (Table 2, Quote 4). Both positive and negative results were described as acting to “reassure” prescribing decisions. Positive results supported clinicians’ views that prescribing an antibiotic was likely to be beneficial, and negative results provided reassurance to withhold or stop antibiotics when the clinician previously was uncertain (Table 2, Quotes 4 and 5).

#### Positive results were valuable in supporting antibiotic choice and stewardship

Most clinicians believed that positive Pneumonia Panel results (i.e., detection of bacterial pathogens) would improve antibiotic choice and AMS. Positive results were often considered to “*confirm”* a HAP/VAP (Table 2, Quotes 6 and 7), and clinicians described using the specific results to choose appropriate antibiotic cover for the organism(s) detected and their resistance determinants (Table 2, Quote 8). Some clinicians considered Pneumonia Panel results as enabling an earlier narrow-spectrum antibiotic prescription and thus facilitating local AMS (Table 2, Quote 8).

#### Results aid differential diagnosis for patients with COVID-19

This study was conducted during the winter 2020/2021 wave of the COVID-19 pandemic, and, in total, around one-third of the patients recruited to INHALE’s RCT had underlying SARS-CoV-2 infection. Participants who treated adult critical-care patients with COVID-19 reported difficulty in distinguishing between virus-induced inflammation and secondary bacterial infection. Adult patients with COVID-19 often had clinical presentations consistent with bacterial infection despite having none; moreover, some COVID-19 treatments (e.g., tocilizumab) rendered certain inflammatory markers unreliable (Table 2, Quote 9) (28). Some clinicians described potentially conflicting treatments for inflammation (i.e., giving immunosuppressives, principally steroids; reconsidering antibiotics) and secondary bacterial infections (i.e., giving antibiotics; avoiding immunosuppressives), but felt quick decision-making was essential because these patients could deteriorate quickly (Table 2, Quote 10).

During the first wave of the pandemic (Spring 2020, before the start of this study), ICU patients with COVID-19 frequently received broad-spectrum antibiotics and some clinicians questioned whether these were necessary (Table 2, Quote 11).

Most participants valued the availability and rapidity of Pneumonia Panel results’ during the pandemic and used the results to aid decisions around antibiotics and high-dose steroids. They especially welcomed having positive results for refining inactive or disproportionate therapy, whereas negative results bolstered their confidence in de-escalating or stopping antibiotics and starting steroids (Table 2, Quotes 9 and 11).

### Doubts about the necessity to modify antibiotic prescribing in accordance with rapid molecular test results

#### “Treating the patient, not the result”

Clinicians described cases when they were reluctant to apply rapid diagnostic results to their antibiotic-prescribing decisions. They described that they would still prescribe antibiotics, despite a negative result if they reasonably suspected the patient had clinical indicators of infection, which may require antimicrobial treatment—prioritizing the patient in front of them, “treating the patient, not the result.” (Table 3, Quotes 1–4). Some clinicians also described following their “gut instinct” and the clinical presentation of the patient sometimes over and above guideline recommendations (Table 3, Quote 4).

#### Negative results create dilemmas

The value of negative Pneumonia Panel results (i.e., detecting neither bacteria nor resistance genes) was more nuanced. Some participants interpreted negative results as indicators that a bacterial respiratory infection was unlikely (Table 3, Quotes 5–7) and de-escalated treatment or stopped a broad-spectrum antibiotic in response. However, for some clinicians, negative results created a dilemma when the “clinical picture” appeared at odds with the machine result. For example, negative results were sometimes interpreted as a sign that the source of infection was elsewhere in the body (i.e., non-respiratory) if their patient was clinically deteriorating (Table 3, Quotes 6 and 7).

#### Initial skepticism and unfamiliarity

Many clinicians described an initial skepticism and unfamiliarity with the Pneumonia Panel test, which led to doubts and concerns about applying test results to their prescribing decisions. Some described colleagues as being more averse to new ways of working and more resistant to change (e.g., the introduction of the Pneumonia Panel) (Table 3, Quotes 8 and 9). Others described an unfamiliarity, where they felt they had not yet reasonably had enough exposure or experience of using the machine to develop confidence in using it to guide their prescribing (Table 3, Quotes 10 and 11).

#### Variable knowledge of the tests’ inherent limitations

Many clinicians discussed the inherent limitations of the Pneumonia Panel molecular diagnostic test, including its inability to detect fungal infections, specific bacteria (e.g., *Stenotrophomonas maltophilia*), and certain resistance genes (e.g., AmpC genes). However, these clinicians did not consider these constraints as necessarily prohibitive to the test’s clinical adoption; rather, they recognized that all tests have limitations and valued being aware of and understanding them (Table 3, Quote 12).

Clinicians reported some views that appeared to be based on misunderstandings of the spectrum, performance, and limitations of the Pneumonia Panel test. For example, some were unsure of the Pneumonia Panel’s targets (e.g., holding the misconception that it could detect fungal infections) and consequently were concerned about insufficient therapy to cover such target organisms (Table 3, Quote 13). Some also incorrectly believed that patients must be “off antibiotics” before using the test (Table 3, Quote 14).

#### Respiratory sample unavailability and of uncertain quality

Some clinicians valued the Pneumonia Panel’s ability to use sputum samples in COVID-19 patients, for whom they were less likely to perform bronchoalveolar lavages (BALs). However, others described numerous situations where obtaining lower respiratory tract samples was challenging, limiting the Pneumonia Panel’s potential utility. For example, the test could not be used for patients who were unable to produce the necessary minimum 200 µL of sample (Table 3, Quote 15). Clinicians also described operational factors that precluded sampling. For example, research nurses’ competing demands and difficulty reaching patients in less-accessible locations inhibited sampling (Table 3, Quote 16). Furthermore, during COVID-19 surges, many units had non-ICU doctors treating patients in makeshift ICUs; these physicians were sometimes unaware that the test was available.

Some clinicians highlighted doubts about the consistency and quality of the respiratory samples and the impact of this on result reliability. In the same context, they raised uncertainties about the quality of samples obtained and potential environmental contamination of the device due to its location at the POC (Table 3, Quotes 17 and 18). Some clinicians suspected that BAL-type samples would lead to more accurate results than sputum-like samples due to less contamination from colonizing bacteria from more proximal airways, whereas others questioned the quality of BAL samples (Table 3, Quotes 19 and 20). Many participants would value trial data demonstrating how different sample types affect the molecular diagnostic test’s accuracy.

#### False-positive results encouraging antibiotic overtreatment

Clinicians suspected that the Pneumonia Panel test would detect colonizing bacteria that were not causing harm. They raised concerns that results reporting non-pathogenic bacteria would encourage unnecessary broad-spectrum antimicrobial therapy, especially because molecular diagnostic results were not filtered by microbiologists to remove likely colonizers (Table 3, Quote 21).

The Pneumonia Panel test uses a semi-quantitative assay to indicate the approximate numbers of each bacterial species found, with a range across 10^4^ to >10^7^ copies/mL sample. Some ICU consultants valued this semi-quantitative component as potentially predicting whether detected organisms were likely pathogens; however, others were unsure how to interpret these results (Table 3, Quotes 22 and 23).

#### False-negative results leading to antibiotic undertreatment

Many clinicians were also worried that false-negative results would lead to incorrectly withholding or stopping antimicrobial therapy and highlighted concerns about subsequent patient-related and legal consequences (Table 3, Quotes 24 and 25). Some perceived false-negative results to be of greater concern than false positives, believing the consequences of antibiotic undertreatment to be more severe (and potentially lethal) than those associated with overtreatment (Table 3, Quote 26).

Some clinicians discussed strategies that they implemented to address their uncertainty about negative results. For example, one clinician described repeating the test with a BAL-type sample, others continued antibiotics, monitored the patient, and revisited their decision after 48 hours (Table 3, Quotes 24, 26, and 27).

#### Concerns about how results influence existing antimicrobial stewardship structures and communications

Several clinicians raised concerns about the integration of the device into routine practice. Given concerns of antibiotic overtreatment following colonizer detection, many cautioned that the test should only be used if an infection was reasonably suspected. They predicted that routine use in the absence of reasonably suspected infection might result in overtreatment and—due to limits on the number of samples that could be run concurrently—potentially limit testing for deteriorating patients who potentially might benefit from earlier results (Table 3, Quotes 28 and 29). Concerns were also raised about the communication of results within the AMS team. Consultant intensivists primarily made antibiotic decisions after receiving molecular test results and could contact clinical microbiologists for advice. However, results occasionally became available out-of-hours, and, unless the ICU consultant phoned for input, microbiological input was not received until the following day. Some microbiologists disagreed with antibiotics chosen based on after out-of-hours results and wanted earlier input (Table 3, Quote 30).

Other clinicians interpreted this issue as indicating that communication could and should be improved. Sites developed local methods for sharing results during the INHALE RCT; these included email and WhatsApp as well as discussing them at microbiology ward rounds and/or writing them in patient notes and drug charts. These clinicians recommended integrating Pneumonia Panel results into local patient record systems to facilitate rapid multidisciplinary team access, also ensuring that results would be easily accessible when revisiting past decisions (Table 3, Quotes 31 and 32).

#### Uncertainty about the evidence base for the molecular diagnostic’s clinical usage

Many participants wanted more familiarization with the Pneumonia Panel test to bolster their confidence in its capabilities and their interpretation of its results. Most wanted this familiarization to determine for themselves whether the test’s benefits outweighed its limitations (Table 3, Quote 33).

For some, familiarization would require additional first-hand experience of the test, either as part of the INHALE RCT or in routine usage. Some described that frequent usage (e.g., during the COVID-19 surge) built confidence (Table 3, Quote 34). Clinicians felt familiarization with “real-world” trial results would significantly affect their confidence in the test. These doctors wanted to determine whether the machine’s results are microbiologically accurate and non-inferior to standard laboratory culture (Table 3, Quotes 35 and 36).

## DISCUSSION

This is the first study to examine clinicians’ perceptions of using a rapid molecular microbiology diagnostic, specifically the Pneumonia Panel test, as an aid to their antibiotic prescribing for HAP/VAP in the ICU in practice.

Our analysis identified a number of key attitudes that may have affected the use and impact of rapid diagnostic tests—such as the Pneumonia Panel—in the ICUs participating in the INHALE RCT, corroborating our previous work (16, 17). Most clinicians were convinced by the importance of AMS and acknowledged that Pneumonia Panel test results could facilitate the earlier refinement of antimicrobial therapy. However, the impact of rapid diagnostic test results on individual prescribing decisions (e.g., to guide the initial antibiotic prescription or to swiftly stop broad-spectrum antibiotics) was limited. Many described counterviews, which meant clinicians often felt reluctant to apply test results to their antibiotic-prescribing decisions. For example, “treating the patient, not the result” was described to be a key driver of prescribing behavior, whereby antibiotics would still be prescribed to a sick patient, regardless of the Pneumonia Panel test result because it fits with the clinical picture. Furthermore, some also cited an initial skepticism and unfamiliarity with the test as factors influencing their perceptions and experience using the test in practice to guide their prescribing decisions, describing their confidence in the test needing to be built up.

Consistent with previous research (16, 29–31), clinicians also described a range of concerns that impeded the application of the test result on their prescribing practices. For example, there were concerns about antibiotic undertreatment resulting from false-negative results (e.g., owing to a pathogen or resistance gene being missed), highlighting that this would negatively affect patient care and expose clinicians to legal consequences. Conversely, results detecting non-pathogenic colonizing bacteria would encourage antibiotic over-usage. Clinicians also discussed concerns surrounding the test’s inherent limitations. Some had misapprehensions and misconceptions about its capabilities. Additionally, clinicians were uncertain about respiratory sample quality (e.g., BAL vs sputum sampling)—an issue that applies also for samples sent for routine laboratory culture.

Clinicians’ doubts and concerns meant that recommendations, based on test results, to avoid initial broad-spectrum antibiotic prescriptions or to swiftly curtail broad-spectrum antibiotic treatment early often were not followed. Rather, perceptions that a broad-spectrum antibiotic prescription was necessary to protect both patient and clinician from the adverse consequences of a pathogen not being detected by the Pneumonia Panel resulted in a broad-spectrum prescription or continuation despite the test result, “erring on the side of caution.” Our findings are consistent with previous research suggesting that despite perceiving AMS to be important (32, 33), many clinicians are hesitant to use rapid diagnostics to influence their prescribing decisions. For example, a recent randomized study examining POC tests for suspected pneumonia in Denmark found that these tests did not significantly affect prescriptions of no or narrow-spectrum antibiotics in the first 2 days of admission (34). Furthermore, a retrospective observational study of patients presenting with viral respiratory infections (VRIs) in the US Emergency Departments demonstrated that despite a diagnosis of VRI, 21% of patients were still prescribed antibiotics (30).

Data in this study were collected during varying stages of the COVID-19 pandemic. Clinicians appreciated using these tests during the COVID-19 pandemic to rule in/out bacterial co-infection and to support their decisions about prescribing (or not) antibiotics and high-dose steroids. However, some clinicians also described difficulty obtaining respiratory samples from patients with COVID-19, who often produced insufficient sputum. Although these concerns were in the context of the COVID-19 pandemic, they reflect wider potential barriers to usage.

This study has limitations. First, most participants were ICU consultants (80%), and all four microbiologists interviewed were from teaching or specialist hospitals in London, meaning that our sample may not be representative. Second, we did not evaluate the role of prescriber concerns around the possibility of patients having occult non-pulmonary infections (e.g., from central lines); research is needed to assess these aspects and how they may affect prescribing for the “pneumonia.” Finally, although we recruited participants from a range of English ICUs, clinicians’ beliefs may differ in non-ICU wards, elsewhere in the United Kingdom, and in other countries.

Our work also suggests possible avenues for further research in molecular diagnostics. First, more data are needed on the extent to which different sample types and quality affect result accuracy and clinical outcomes. Second, research should focus on how to distinguish pathogens from colonizers not only using molecular diagnostics but also by standard-of-care culture methods, as this is a general issue for infections at non-sterile body sites such as the respiratory tract.

This study highlights the complexities of clinical decision-making in ICUs. The Pneumonia Panel results were valued in principle, but in many cases, the influence of result on prescribing decision was limited. This was particularly salient when clinicians described a conflict between the data produced by the machine and the complex clinical picture presented by the patient. Our findings highlight that clinicians’ reluctance to apply Pneumonia Panel test results to an initial prescription and/or later de-escalation of antibiotics was often largely driven by a range of factors beyond biomedical data and the guidelines of current evidence-based medicine. Instead, clinicians’ were influenced by their “mindlines,” meaning “collectively reinforced, internalized, tacit guidelines*,*” which are iterative and flexible (35, 36). These “mindlines” are characterized by interactions with patients and colleagues, and clinicians’ “knowledge in practice” and perceptions informed by training and the experiences of themselves and others (e.g., “I’ve been here before and been burned by my decision not to prescribe antibiotics”). Our findings seem to illustrate a tension between guidelines and “mindlines” with implications for how technological approaches to antibiotic stewardship might be applied in practice. Although this study explores clinicians’ specific experiences and perceptions of using the Pneumonia Panel test, the principles and issues surrounding clinicians’ perspectives are likely to be transferrable towards the implementation of many, if not most, new diagnostic technologies in medicine.

The impact of technological and guideline solutions to AMR may be limited if we fail to recognize the impact of clinical “mindlines” on prescribing decisions. Our findings demonstrate that clinicians’ beliefs and emotions are often key drivers of their antibiotic prescribing. Governed by the wish to save lives, doctors ultimately behave in more protective ways than may be objectively necessary. Therefore, the implementation of technological or guideline-based solutions to antimicrobial resistance needs to be behaviorally intelligent, understanding, and connecting with the way in which clinicians think about the problem at hand and respond to it.

### Conclusion

Although most clinicians saw potential for the Pneumonia Panel to support stewardship, the practice of using test results to avoid prescribing a broad-spectrum antibiotic or to stop one early was often overridden by clinicians’ imperative to prescribe a broad-spectrum antibiotic “just-in-case” as a mechanism to protect the patient, “erring on the side of caution.” Clinicians described cases where antibiotics would be prescribed for a sick patient regardless of the Pneumonia Panel test result because in their opinion, that fits with the clinical picture, *“*treating the patient, not the result*.”* The data in this study identify a tension between evidence-based medicine and the art of medicine, acknowledging the human-to-human nature of antibiotic prescribing in ICU. Specifically, our findings suggest clinicians’ “mindlines”—inclusive of their previous experiences and those of their colleagues, “knowledge in practice” and, importantly, the patient in front of them—are key drivers of their antibiotic prescribing, often over and above hospital prescribing guidelines and the results of molecular diagnostics. The optimal implementation of the latter tests in practice, therefore, requires a “technology plus” approach, acknowledging the challenges clinicians face when applying technological solutions to the care of individual patients.

## SUPPLEMENTAL MATERIAL

File S1 aac.01156-24-s0001.docx Organisms detected by the Pneumonia Panel test.

File S2 aac.01156-24-s0002.docx Research checklist.

File S3 aac.01156-24-s0003.docx Interview guide.

ASM does not own the copyrights to Supplemental Material that may be linked to, or accessed through, an article. The authors have granted ASM a non-exclusive, world-wide license to publish the Supplemental Material files. Please contact the corresponding author directly for reuse.
