# Supplementary material for: Guidelines vs mindlines: a qualitative investigation of how clinicians’ beliefs influence the application of rapid molecular diagnostics in intensive care
Source: Antimicrob Agents Chemother. 2025 Feb 5;69(3):e01156-24. doi: 10.1128/aac.01156-24 (PMC11881570; doi:10.1128/aac.01156-24)
Supplement: File S1 — Organisms detected by the Pneumonia Panel test. [file aac.01156-24-s0001.docx]

**Supplementary Material S3: Interview Guide**

As you probably know, the aim of this project is to study hospital doctors’ experiences of using the BioFire device and prescribing algorithm with HAP and VAP patients. We’d like to understand whether these tools are useful for you in practice.

- Have you had time to read the information letter?

Then you are aware that this interview will be tape recorded, transcribed verbatim, and then analysed by a team of scientists. We guarantee you confidentiality and you can withdraw from the study at any time until it has been published.

- Do you have any questions about what I have just said?
- Could you please sign the consent form?

Thank you so much for participating. I’ll first ask some questions about BioFire results and then move onto questions about the prescribing algorithm.

*Topic: Experience with BioFire result and algorithm recommendations*

- What are your views about the BioFire?
- Could you tell me about a time when you made an antibiotic decision with BioFire results?
  - How did the algorithm feed into your decision-making?
  - How did other clinical staff feed into your decision-making?
- What would you have done if you didn’t have access to the BioFire?
- What would you have done if the BioFire results had been [positive/negative; as appropriate]?
- Can you compare how you make antibiotic choices with and without the BioFire?
- Can you compare how you use the BioFire with COVID and non-COVID patients? Are there any major differences?

*Topic: Barriers/facilitators to incorporating BioFire results into antibiotic decision-making*

- Which part of the BioFire results do you find the most useful? Why?
  - Quantitative vs. qualitative components of test; resistance genes; atypical bacteria; viruses
- Do you have any concerns with the BioFire results?
- How do your colleagues feel about BioFire results? [intensivists; microbiologists]
- Does having access to BioFire affect your likelihood to prescribe empiric antibiotics?

*Topic: Barriers/facilitators to compliance with prescribing algorithm recommendations*

- Do you find it easy or difficult to adhere to algorithm recommendations? Why?
  - To what extent do you have confidence in the algorithm’s recommendations?
  - Do you have any concerns with the algorithm?
- Do your colleagues comply with the algorithm recommendations? Why/why not?

Is there anything we haven’t covered that you’d like to talk about?

Thank you so much for your time!
