# Supplementary material for: Guidelines vs mindlines: a qualitative investigation of how clinicians’ beliefs influence the application of rapid molecular diagnostics in intensive care
Source: Antimicrob Agents Chemother. 2025 Feb 5;69(3):e01156-24. doi: 10.1128/aac.01156-24 (PMC11881570; doi:10.1128/aac.01156-24)
Supplement: File S3 — Interview guide. [file aac.01156-24-s0003.docx]

**Supplementary Material S1: Organisms detected by the BioFire FilmArray Pneumonia Panel**

| **Bacteria (semi-quantitative)** | **Viruses** |
| --- | --- |
| *Acinetobacter calcoaceticus-baumannii* complex  *Enterobacter cloacae* complex  *Escherichia coli*  *Haemophilus influenzae*  *Klebsiella aerogenes*  *Klebsiella oxytoca*  *Klebsiella pneumoniae* group  *Moraxella catarrhalis*  *Proteus* spp.  *Pseudomonas aeruginosa*  *Serratia marcescens*  *Staphylococcus aureus*  *Streptococcus agalactiae*  *Streptococcus pneumoniae*  *Streptococcus pyogenes* | Adenovirus  Coronavirus (NOT SARS-CoV-2)  Human Metapneumovirus  Human Rhinovirus/Enterovirus  Influenza A  Influenza B  Parainfluenza Virus  Respiratory Syncytial Virus |
| **Atypical bacteria (qualitative)** | **Antibiotic resistance genes** |
| *Chlamydia pneumoniae*  *Legionella pneumophila*  *Mycoplasma pneumoniae* | Methicillin resistance:   - *mec*A/C and MREJ   Carbapenemases:   - KPC - NDM - Oxa-48-like - VIM - IMP   ESBL:   - CTX-M |

Source: Biomérieux. The BioFire® FilmArray® Pneumonia (PN) Panel. 2021. Available at: biofiredx.com/products/the-filmarray-panels/filmarray-pneumonia/. Accessed 17 February 2021.
